# Intestinal Parasitic Infections and Associated Risk Factors Among Children in an Internally Displaced Center (IDC) in Kosti City of the White Nile State, Sudan

**DOI:** 10.1155/japr/5542680

**Published:** 2025-04-14

**Authors:** Abdelhakam G. Tamomh, Mohamed Osman Ali, Williams Walana, Neima Mhmoud Mohammed, Ibrahim M. Hassan, Manasik Elghali Hamed, Mohamed JamalAldein Alkab, Ghalia Ibrahim Mohammed, Einas Abohurairh Albashir, Rayan Obeid Mahmoud, Malaz Salah Ahmed

**Affiliations:** ^1^Department of Parasitology and Medical Entomology, Faculty of Medical Laboratory Sciences, White Nile University, Kosti, Sudan; ^2^Department of Parasitology and Medical Entomology, Faculty of Medical Laboratory Sciences, University of El Imam El Mahdi, Kosti, Sudan; ^3^Department of Clinical Chemistry, Faculty of Medical Laboratory Sciences, University of El Imam El Mahdi, Kosti, Sudan; ^4^Department of Clinical Microbiology, School of Medicine, University for Development Studies (UDS), Tamale, Ghana; ^5^Department of hematology, Faculty of Medical Laboratory Sciences, White Nile University, Kosti, Sudan

**Keywords:** children, intestinal parasites, prevalence, Sudan, war

## Abstract

**Background:** Intestinal parasitic infections (IPIs) are considered a global health problem, especially in developing countries such as Sudan. Over three million people have been displaced in Sudan due to civil war, and children are severely affected. However, there is limited data regarding IPIs among children in internally displaced centers (IDCs) in the White Nile State, especially in Kosti City. Therefore, this study aimed to determine the prevalence of IPIs and the associated risk factors among Sudanese children in an IDC in Kosti City, White Nile State, Sudan.

**Methods:** A cross-sectional study was conducted among 508 children at the IDC, and 508 corresponding stool samples were collected. Parents and guardians of children aged 1–10 were contacted randomly to voluntarily enroll their wards in the study. The sample collection period was from June 15th to November 15th, 2023. The stool samples were examined using direct wet mount and formal ether concentration techniques.

**Results:** The overall prevalence of IPIs among children of the IDC was 33.9% (172/508). Specifically, the prevalence of intestinal parasites (IPs) among the children were *Entamoeba histolytica* (6.7%), *Giardia lamblia* (25.0%), and *Hymenolepis nana* (2.2%). Age-wise, the proportion of IPIs among children (1–5 years) was 29.8% (59/198), and those 6–10 years were 40.3% (125/310), *p* < 0.05. IPIs among males (32.1%) were less than females (34.9%), *p* > 0.05.

**Conclusion:** The study concluded a relatively high prevalence of IPIs among children in IDC in Kosti City, White Nile State, Sudan. Therefore, a swift public intervention plan is needed, such as improving hygiene and sanitation, health education, and periodic screening and deworming of the affected population, particularly children.

## 1. Introduction

Internally displaced persons (IDPs) are persons who have been forced to leave their homes of living due to conflicts, disasters, and violence and who have not crossed their country's border. Globally, there are 71.1 million IDPs, with more than half in Sub-Saharan Africa by the end of 2022, according to the Internal Displacement Monitoring Center (IDMC). Additionally, more than 56% of the internally displaced individuals are children. Most of the IDPs live in host communities, sometimes in universities, schools, churches, and mosques [1, 2]. Population displacements always affect general health systems. IDPs are the most of the forced migrants, and the mortality increased mainly among children and pregnant women [3, 4].

In Sudan, from 15 April 2023, armed clashes erupted between the Sudanese Armed Forces (SAF) and the Rapid Support Forces (RSF). As a result of the military clashes, over 3.7 million individuals have been internally displaced, and patches of displacement have been observed in over 6159 areas across all of Sudan's 18 states. The highest proportions of IDPs have been observed around River Nile (12.45%), South Darfur (11.09%), East Darfur (10.82%), Al Jazirah (7.92%), Northern (7.82%), and North Darfur (7.34%). From the total IDP reported, more than 120,338 individuals are located across Blue Nile, El Gadarif, Kassala, North Kordofan, South Kordofan, Northern, Red Sea, River Nile, and White Nile states [5, 6]. One of the common health challenges among people displaced by war and natural disasters across the globe is exposure to various forms of infections, and the case of Sudan is no exception. Due to unhygienic conditions in IDP camps, bacteria, parasitic, and viral infections of the intestinal tract are common, particularly among children.

Intestinal parasitic infections (IPIs) are major health problems in many developing countries such as Sudan, particularly among preschool and school children [7–10]. Morbidity and mortality associated with pathogenic intestinal parasites (IPs) including protozoa like *Entamoeba histolytica (E. histolytica)*, *Giardia lamblia (G. lamblia)*, *Cryptosporidium* spp., *Balantidium coli*, and *Dientamoeba fragilis* and helminths such as *Ascaris lumbricoides*, *Trichuris trichiura*, and *Strongyloides stercoralis* are observed in both developing and underdeveloped countries [8, 9]. IPIs may be asymptomatic or may present with clinical features such as abdominal pain, cramps, nausea, vomiting, diarrhoea, weight loss, intestinal obstruction, anaemia, poor appetite, worsening of nutritional status, and impaired physical development [11, 12].

IPIs are more prevalent among school-aged children as reported by numerous studies [8, 13]. Displaced children living in internally displaced centers (IDCs) are at risk of infection with different types of pathogens, including parasites, due to the living conditions in addition to differences within and among camps, such as large family size, lack of clean water, and poor personal hygiene with weak nutritional status, especially for children. These conditions generally predispose one to varied infections, especially in vulnerable groups like children. It is scarce to identify studies focusing on IPs among children in IDCs, particularly in the Sudanese population. It is for this reason that the goal of this study was to determine the prevalence of IPIs among Sudanese children in IDCs at Kosti City, White Nile State, Sudan.

## 2. Methods

### 2.1. Study Design, Setting, and Population

**Figure 1:**
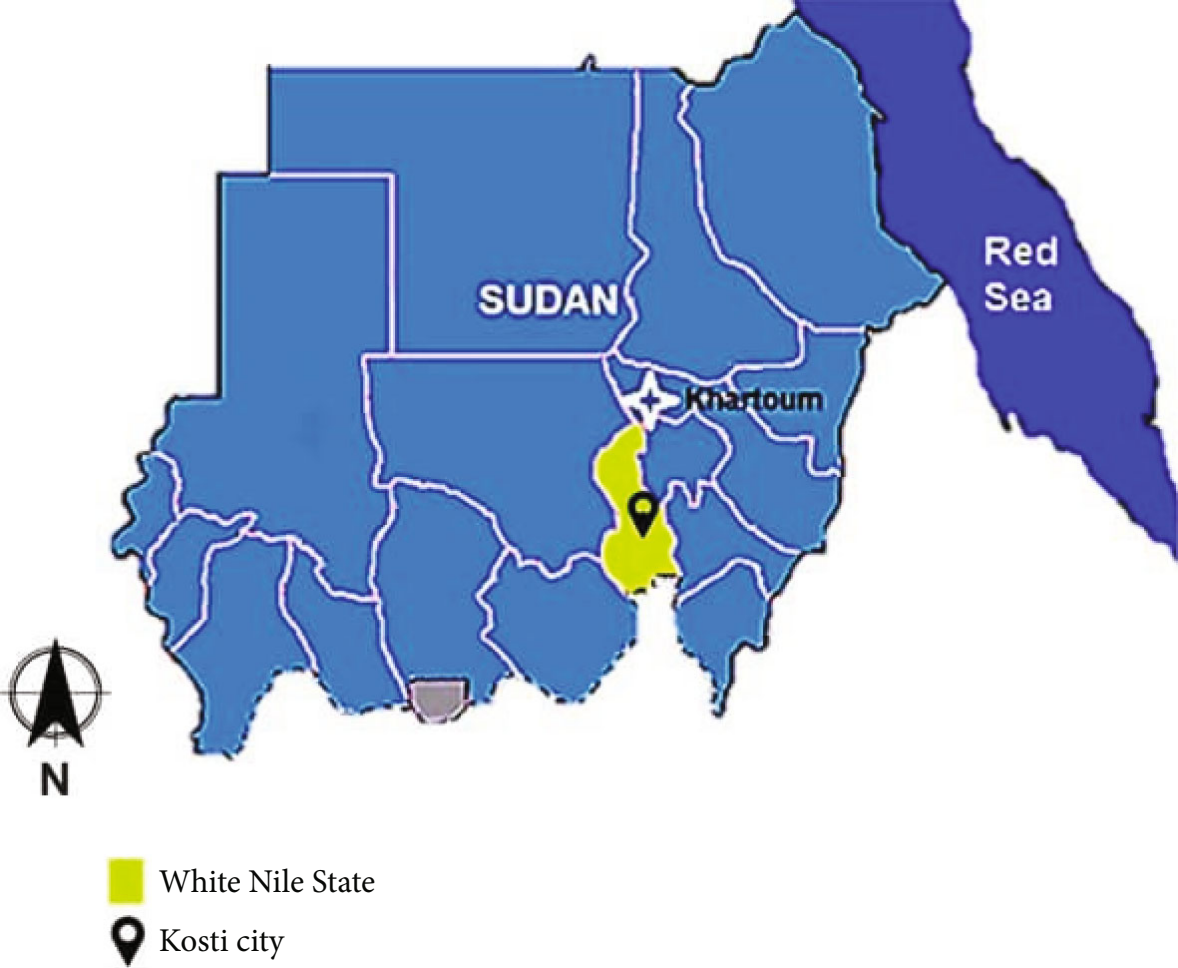
Map of Kosti City [14].

A cross-sectional study was conducted at the University of El Imam El Mahdi Girls campus, which houses IDPs at Kosti City. The Camp is located in the middle of Kosti City, one of the major cities in White Nile State, that lies south to Khartoum, the capital of Sudan, and is located on the western bank of the White Nile River. The location of Kosti City lies between longitude 13.12 and 13.40 E and between latitude 13.39 and 32.45 N, and at altitude 382 m above sea level (Figure 1). The climate has a long rainy season which lasts for 6 months (April–September) [14–16]. The study included both sexes of preschool and school-aged children in the camp. A total of 508 children resident in the camp were included during the study period, from June 15th, 2023, to November 15th, 2023. Children over 10 years old and those not resident in the IDC were exempted from the study.

### 2.2. Ethical Consideration

The protocol for the present study was approved by the institutional ethics committee of the Faculty of Medical Laboratory Sciences, White Nile University, Kosti City, Sudan (No. EA.FMLS.WNU-2023-7). The study was conducted in compliance with the Helsinki Declaration for Research. Before stool specimens were collected, consent was taken from the coordinator and other relevant authorities in the center, including the parents/guardians of the children.

### 2.3. Sample Collection and Processing

Parents and guardians at the IDC with children aged 1–10 years were contacted randomly to enroll their wards for the study voluntarily. For those who gave consent, stool samples were collected from their children for the study. All samples were collected in a sterile, dry, clean stool container, labeled with a name and serial number, checked for ideal stool quantity, and examined in the Parasitology Laboratory at the Faculty of Medical Laboratory Sciences, White Nile University. A structured questionnaire was used to collect the children's demographic data and some camp practices.

### 2.4. Direct Wet Mount Method

Each stool sample was subjected to a direct saline wet mount procedure to identify IPs microscopically, as described in previous studies [17, 18]. Briefly, each stool sample was uniformly mixed with a wooden applicator and about 0.1 g of stool was emulsified in a drop of physiological saline on a glass slide, which was covered with a cover slip. Three technicians independently assessed each stool microscopically, and the results synchronized.

### 2.5. Formal Ether Concentration Sedimentation Method

From each well-mixed stool specimen, a small size was taken using a wooden stick and processed following standard instructions [17] . About 1 g of stool was dropped into a clean container, and 7 mL of 10% formol saline was added, emulsified, and sieved using a fine sieve. The sieved stool sample was transferred to a 15-mL centrifuge tube, 2 mL of diethyl ether added, shaken gently for a few seconds, and centrifuged. The fecal debris was released from the top layer, and the supernatant was discarded. After that, the sediment was stained with iodine, mixed by means of a Pasteur pipette and transferred to a microscopic slide, covered with cover glass, and examined microscopically using 10x and 40x objectives.

### 2.6. Data Analysis

Data collected was analyzed using the IBM-Statistical Package for Social Sciences (SPSS) version 21. Frequency and percentages were reported for descriptive statistics. Pearson's chi-square (*χ*^2^) was used to evaluate the association between variables. Bivariate analysis was used to identify potential risk factors. *p* values < 0.05 were considered significant for all statistical analyses.

## 3. Results

### 3.1. Characteristics of Participated Children in IDC

Then, 508 stool samples were obtained from 508 children at the University of El Imam El Mahdi IDC in Kosti City. The children's ages ranged from 1 to 10 years old, with a mean age of 5.9 (SD = 2.38). More than half (61%) of the children were within the group 6–10 years. The number of females (68.0%) was higher than males (32.0%). Regarding the sources of water in the IDC, most inhabitants depended on donkey cart water (85.0%), and the rest (15.0%) used pipe water, as expressed in Table 1.

### 3.2. Prevalence of IPs Among Participated Children in IDC

The overall prevalence of IPIs among the participating children was 33.9% (172/508). Three (3) species of IPs were detected and identified. *G. lamblia* was registered in 127 children and representing 25.0%, *E. histolytica* was recorded in 34 children and representing 6.7%, and *Hymenolepis nana* (*H. nana*) was registered among 11 children representing 2.2%. There was a high difference in IPs' prevalence among participated children (*p* < 0.001), as expressed in Table 2.

### 3.3. IPIs and Associated Risk Factors Among Participated Children in IDC

As expressed in Table 3, IPI prevalence was higher among children aged 6–10 years compared to children aged 1–5 years, with statistically significant association (*χ*^2^ = 7.203, OR = 1.666, 95% CI: 1.146–2.424, *p* = 0.007). According to gender, the infection rate was higher among females than males, but no statistically significant association (*χ*^2^ = 0.882, OR = 0.418, 95% CI: 0.603–1.291, *p* = 0.508). The IPI prevalence was higher among children with diarrhoea than children without diarrhoea, with a statistically significant association (*χ*^2^ = 187.37, OR = 31.028, 95% CI: 0.603–1.291, *p* < 0.001). Regarding the sources of water in the IDC, most of them depended on water supplied from the White Nile River by donkey carts 432 (85%) and the rest, 76 (15%) used pipe water, which 19 (25%) and 153 (36.2%) of them were infected, respectively (*χ*^2^ = 3.131, OR = 0.608, 95% CI: 0.349–1.059, *p* = 0.077). There was no statistically significant association between the IPIs and the water source. Regarding the habit of hands washing before eating, 270 (53%) always did so, and 238 (47%) occasionally did, of which 75 (27.7%) and 97 (40.8%) were infected, respectively. There was an association between washing hands habit before eating and IPIs among the children (*χ*^2^ = 9.515, *p* = 0.002). Among all children in this study, 141 (27.8%) had a habit of eating raw meat and vegetables, and 40 (28.4%) of them were infected, while 367 (72.2%) have not a habit of eating raw meat and vegetables, with 133 (36.2%) of them infected. There was no significant association between IPIs and eating raw meat and vegetable habits (*χ*^2^ = 2.626, OR = 0.705, 95% CI: 0.472–1.77, *p* = 0.105).

## 4. Discussion

IPIs are still a significant health problem in developing regions around the world, and the case of Sudan is no different [8, 9]. The prevalence of IPIs in an area informs the health, sanitation, living conditions, and public health status of the inhabitants. To the best of our knowledge, this is a maiden study on Sudanese children in IDC in the Kosti City of the White Nile State in Sudan.

In our current finding, the overall prevalence of IPIs among children in the IDC was 33.9% (172/508). Our finding was higher than that of the results obtained from Kirkuk province/Iraq [19] (14.4%), Duhok province/Iraq [20] (5.83%), and Iran [11] (9.8%), but lower than the results obtained from Central Sudan [13] (90.4%), Nigeria [1] (89.6%), Pakistan [21] (65.33%), and southern Ethiopia (67.4%) [22]. These differences in prevalence might be related to variations in children's ages, geographical and environmental conditions, sample size, time of sample collection, experimental techniques, poor personal hygiene, and sources of drinking water.

The study revealed that the prevalence of IPIs was higher among females (110 (34.9%)) than males (62 (32.1%)), but statistically, it was not significant (*p* > 0.05). This observation is in agreement with the result from Nigeria [1] and in disagreement with that obtained from Duhok province in Iraq [20], southern Ethiopia [22], and eastern and central Sudan [7, 13]. This study could not explain why females were more infected in the current study, indicating the need for an expanded future study given the variation in literature.

In the present study, the high prevalence of IPIs was statistically significant among the age group 6–10 years compared to the age group 1–5 years. This result is in line with that obtained from Iraq and Nigeria [1, 19]. The variations in the IPI rate could be attributed to the more active nature of children, playing in the contaminated ground, and other factors such as the state of the IDC and the environmental and sanitary habits the children have been exposed to.

In the current study, the prevalence of IPIs was statistically more significant among children with diarrhoea than among children without diarrhoea. This finding supports the reports of previous studies [15, 18]. Our analysis revealed that there was an association between washing hands before eating and IPIs among children in IDC. This result is in agreement with the finding from Nigeria [1]. This finding suggested that children in IDC acquired IPI from direct contact with infected soil, personal hygiene, and the crowded nature of children in the IDC.

In our present study, three species of IPs were identified. *G. lamblia* was registered in 127 children representing 25%, *Entamoeba histolytica* was recorded in 34 children and representing 6.7%, and *H. nana* was registered among 11 children representing 2.2%. There were significant differences in intestinal parasite prevalence among participating children (*p* < 0.001). Similar findings were gained from Pakistan, Iran, and Nigeria [1, 11, 21].

Contaminated water has been strongly linked to IPIs. However, the current study did not establish any such association. Possibly, the water from the donkey cart (from the White Nile River) is not used as drinking water. The user may also have processed it before use, either by boiling, chlorination, or filtration. Our study could not capture the sources of drinking water.

## 5. Conclusion

The study concludes that the overall prevalence rate of IPIs among children in IDC at Kosti City was 33.9%. IPs were more common in diarrhoeal stools. Therefore, a swift public health intervention plan, such as improving hygiene and sanitation, health education, and periodic screening and deworming of the affected population, is needed.

### 5.1. Limitation

The study duration coincided with the rainy season, which could have influenced the outcomes observed. It is unclear whether the children contracted the infection at the camp or if they were infected before visiting the camp. Prescreening for infections before admission to the camp could help identify the infections acquired. Application of molecular techniques may increase the prevalence of IPs in this study. Data regarding detailed environmental or socioeconomic factors that could potentiate the infections were not captured, limiting linkages to the risk factors identified in this study.

## Figures and Tables

**Table 1:** Demographic characteristics of participated children in IDC in Kosti City, White Nile State, Sudan, 2023.

**Characteristic**	**Frequency**	**%**
Age group (years)		
1–5	198	39
6–10	310	61
Sex		
Male	193	38
Female	315	62
Water sources		
Pipe water	76	15
Donkey cart (from the White Nile River)	432	85

**Table 2:** Prevalence of IPs among children in IDC in Kosti City, White Nile State, Sudan, 2023.

**Intestinal parasites identified**	**No. of positive** **n** ** (%)**	**No. of negative** **n** ** (%)**	**p** ** value**
*Giardia lamblia*	127 (25.0)	381 (75.0)	< 0.001
*Entamoeba histolytica*	34 (6.7)	474 (93.3)	< 0.001
*Hymenolepis nana*	11 (2.2)	497 (97.8)	< 0.001
Overall IPIs	172 (33.9)	336 (66.1)	< 0.001

**Table 3:** Sociodemographic risk factors and IPI association among children at IDC in Kosti City, White Nile State, Sudan, 2023.

**Factors**	**Infected** **n** ** (%)**	**Noninfected** **n** ** (%)**	**χ** ^2^	**OR**	***95% CI***	**p**
Age (years)						
1–5	59 (29.8)	117 (59.1)	7.203	1.666	(1.146–2.424)	0.007
6–10	125 (40.3)	219 (70.6)				
Sex						
Male	62 (32.1)	131 (67.9)	0.882	0.418	(0.603–1.291)	0.518
Female	110 (34.9)	205 (65.1)				
Diarrhea						
Yes	159 (62.6)	95 (37.4)	187.37	31.028	(0.603–1.291)	< 0.001
No	13 (5.1)	205 (94.9)				
Water sources						
Pipe water	19 (25)	57 (75)	3.131	0.608	(0.349–1.059)	0.077
Donkey cart (from the White Nile River)	153 (35.4)	279 (64.6)				
Washing of hands before eating						
Always	75 (27.7)	195 (72.3)	9.515	0.559	(0.386–0.810)	0.002
Occasionally	97 (40.8)	141 (59.2)				
Eating raw meat and vegetable						
Yes	40 (28.4)	101 (71.6)	2.626	0.705	(0.472–1.77)	0.105
No	133 (36.2)	234 (63.8)				

## Data Availability

The data that support the findings of this study are available on request from the corresponding author. The data are not publicly available due to privacy or ethical restrictions.
